# Degradation of methylation signals in cryopreserved DNA

**DOI:** 10.1186/s13148-023-01565-y

**Published:** 2023-09-11

**Authors:** Ning Yuan Lee, Melissa Hum, Guek Peng Tan, Ai Choo Seah, Patricia T. Kin, Ngiap Chuan Tan, Hai-Yang Law, Ann S. G. Lee

**Affiliations:** 1 Division of Cellular and Molecular Research, National Cancer Centre Singapore, 30 Hospital Boulevard, Singapore, 168583 Singapore; 2 DNA Diagnostic and Research Laboratory, KK Women’s and Children’s Hospital, 100 Bukit Timah Rd, Singapore, 229899 Singapore; 3 SingHealth Polyclinics, 167 Jalan Bukit Merah, Singapore, 150167 Singapore; 4 SingHealth Duke-NUS Family Medicine Academic Clinical Programme, Duke-NUS Medical School, 8 College Road, Singapore, 169857 Singapore; 5 SingHealth Duke-NUS Oncology Academic Clinical Programme (ONCO ACP), Duke-NUS Medical School, 8 College Road, Singapore, 169857 Singapore; 6 Department of Physiology, Yong Loo Lin School of Medicine, National University of Singapore, 2 Medical Drive, Singapore, 117593 Singapore

**Keywords:** Methylation, Blood, Cryopreservation, Storage, Buffy coat, DNA

## Abstract

**Background:**

Blood-based DNA methylation has shown great promise as a biomarker in a wide variety of diseases. Studies of DNA methylation in blood often utilize samples which have been cryopreserved for years or even decades. Therefore, changes in DNA methylation associated with long-term cryopreservation can introduce biases or otherwise mislead methylation analyses of cryopreserved DNA. However, previous studies have presented conflicting results with studies reporting hypomethylation, no effect, or even hypermethylation of DNA following long-term cryopreservation. These studies may have been limited by insufficient sample sizes, or by their profiling of methylation only on an aggregate global scale, or profiling of only a few CpGs.

**Results:**

We analyzed two large prospective cohorts: a discovery (*n* = 126) and a validation (*n* = 136) cohort, where DNA was cryopreserved for up to four years. In both cohorts there was no detectable change in mean global methylation across increasing storage durations as DNA. However, when analysis was performed on the level of individual CpG methylation both cohorts exhibited a greater number of hypomethylated than hypermethylated CpGs at *q*-value < 0.05 (4049 hypomethylated but only 50 hypermethylated CpGs in discovery, and 63 hypomethylated but only 6 hypermethylated CpGs in validation). The results were the same even after controlling for age, storage duration as buffy coat prior to DNA extraction, and estimated cell type composition. Furthermore, we find that in both cohorts, CpGs have a greater likelihood to be hypomethylated the closer they are to a CpG island; except for CpGs at the CpG islands themselves which are less likely to be hypomethylated.

**Conclusion:**

Cryopreservation of DNA after a few years results in a detectable bias toward hypomethylation at the level of individual CpG methylation, though when analyzed in aggregate there is no detectable change in mean global methylation. Studies profiling methylation in cryopreserved DNA should be mindful of this hypomethylation bias, and more attention should be directed at developing more stable methods of DNA cryopreservation for biomedical research or clinical use.

**Supplementary Information:**

The online version contains supplementary material available at 10.1186/s13148-023-01565-y.

## Background

DNA methylation shows great promise as a blood-based biomarker for a variety of diseases [1–4]. As analyses of DNA methylation become more sophisticated and prediction models increase in complexity, there is an increasing demand for larger sample sizes. To meet this demand, biobanks or individual laboratories collect and cryopreserve many samples [5]. These samples may remain in storage for long durations, often so they can be sent with subsequently collected samples to be processed in batch, or to be stored as additional aliquots for future analysis.

Importantly, long-term storage may introduce technical biases in the measurement of DNA methylation profiles. These biases can influence increasingly popular “black box” machine learning algorithms [6] or produce false results in sensitive techniques such as methylation-specific polymerase chain reaction (MS-PCR) [7]. Indeed, the degradation of DNA methylation signals has well-known consequences on the analysis of ancient DNA, where the deamination of 5-methylcytosine to thymine introduces false C → T substitutions in ancient DNA sequences [8]. Although these changes in ancient DNA were observed after thousands of years in natural cryopreservation, they may not require thousands of years to occur.

However, it is not clear the extent to which cryopreservation affects human DNA methylation profiles at the time scales of laboratory cryopreservation. Several studies of DNA cryopreserved up to a couple of decades have shown minor decreases in global methylation associated with cryopreservation [9, 10]. Other studies have found no effect [11, 12], while one study has reported a contrary increase in methylation [13]. Since most of these studies profile global or mean methylation across many CpGs or choose a small set of CpGs or gene regions to profile, it is also unclear if certain CpGs are more prone to the effect of cryopreservation than others.

To address these questions, we present here the largest-to-date study on the effect of long-term cryopreservation on both global methylation profiles as well as individual CpGs. We found no detectable change at up to 50 months of cryopreservation when methylation was analyzed on a global scale, but there was a clear bias toward hypomethylation when methylation was analyzed at the level of individual CpGs. Furthermore, CpGs near but not at CpG islands were more likely to be hypomethylated. These results have important implications in the design of methylation analyses utilizing cryopreserved DNA samples, especially for high-sensitivity analyses or “black-box” algorithms.

## Results

### Cohort characteristics

**Fig. 1:**
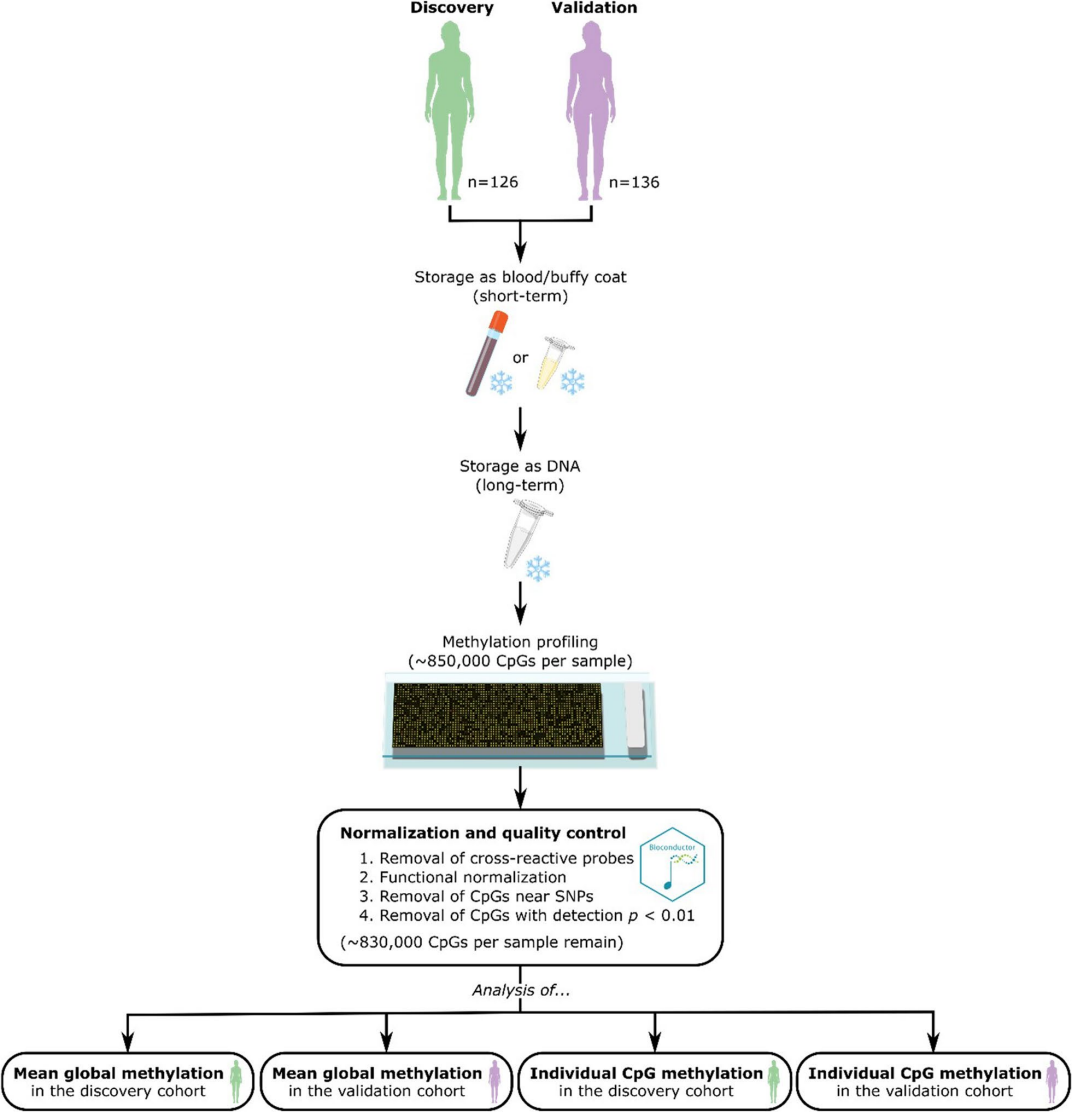
Overview of the study design

We profiled methylation from DNA extracted from the peripheral blood samples of two cohorts of non-cancer volunteers, for discovery (*n* = 126) and validation (*n* = 136) (Fig. 1). Subjects in the discovery cohort were older than those in the validation cohort (median age of 48.0 years and 34.6 years, respectively). All subjects in both discovery and validation cohorts were of self-reported Chinese ethnicity. For the discovery cohort, buffy coats were isolated from blood within two hours of blood draw and stored at -20°C for up to 3 months (median storage duration as buffy coat = 20 days); whereas for the validation cohort, peripheral blood samples were stored at 4°C for up to five days only (“storage duration as blood/buffy coat”). Then, DNA was extracted from each blood/buffy coat sample and cryopreserved (“storage duration as DNA”) until library preparation and methylation profiling. DNA samples from the discovery cohort were stored for a longer duration than those from the validation cohort (median duration in storage of 44.4 months and 21.0 months, respectively) (Table 1). Since samples were stored as extracted DNA for much longer than as pre-extraction blood/buffy coat and it is only known that validation cohort samples were stored as blood at 4°C for five days or less, our analysis focuses on the association between storage duration as DNA and methylation profiles.

**Table 1:** Subject and sample characteristics of the discovery and validation cohorts

	Discovery (*n* = 126)	Validation (*n* = 136)
*Subject characteristics*		
Median age, years (range)	48.0 (38.1–71.5)	34.6 (19.0–44.7)
Self-reported ethnicity		
Chinese	126 (100%)	136 (100%)
*DNA sample characteristics*		
Median storage duration as blood	≤ 2 hours^a^	≤ 5 days^b^
Median storage duration as buffy coat at − 20 °C, days (range)	20 (1–77)	N/A^c^
Median storage duration as DNA at − 20 °C, months (range)	44.4 (33.5–50.1)	21 (15.0–38.0)^d^

^a^Buffy coats were isolated from discovery cohort blood samples within two hours

^b^No detailed information is available for the duration of storage as blood for the validation cohort, except that all blood samples were extracted for DNA within five days

^c^DNA was extracted directly from whole blood using an automated system

^d^For the validation cohort only, this duration includes initial storage at 4°C for up to two months

To ensure independence between discovery and validation phases, the discovery and validation cohorts were preprocessed and filtered for quality control separately. We removed cross-reactive probes, applied functional normalization, removed CpGs near SNPs, and lastly removed poorly detected CpGs. After quality control, there were analyzable data from 830,545 CpGs in the discovery cohort and 830,0551 CpGs in the validation cohort. All statistical tests were performed separately and independently on discovery and validation cohorts so that the differences between cohort characteristics do not confound the analysis. The key differences were that the discovery cohort was stored as buffy coat prior to extraction while the validation cohort was not, and the validation cohort was stored at 4°C for up to two months after DNA extraction before being moved to − 20°C while the discovery cohort was stored at − 20°C immediately after DNA extraction (Table 1).

### Mean global methylation

**Fig. 2:**
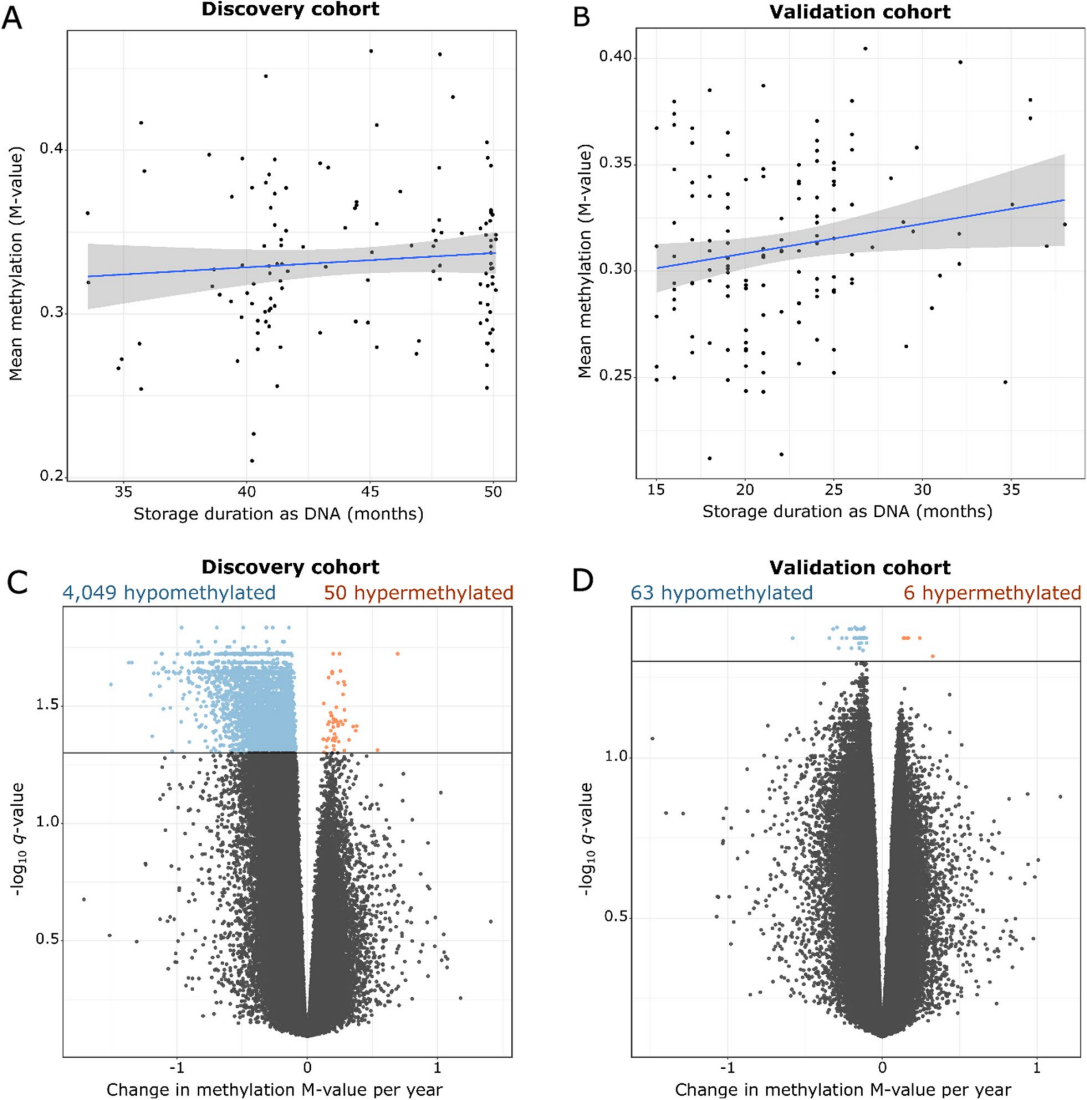
Mean global methylation per sample versus storage duration as DNA **A** for the discovery cohort and **B** the validation cohort; the change in methylation beta-value per year of storage as DNA and their associated *q*-values for **C** the discovery and **D** validation cohorts, where the horizontal line represents the *q*-value < 0.05 threshold

We did not observe any association between mean global methylation and up to 50 months of storage duration as DNA. Mean global methylation was quantified as the mean methylation M-value for all CpGs within a sample. In the discovery cohort, mean global methylation showed no evidence of change across increasing storage durations as DNA (*p* = 0.360) (Fig. 2A), even after controlling for the covariates age, blood cell type composition, and storage duration as buffy coat (*p* = 0.292). In the validation cohort, there was some evidence for an increase in mean global methylation across increasing storage durations as DNA (*p* = 0.030), but not after controlling for the age and cell type composition (*p* = 0.140) (Fig. 2B, Table 2).

**Table 2:** Results for the robust linear regression for mean global methylation. The coefficient estimates for storage durations and age represent the change in methylation M-value per year

Term	Discovery cohort (n = 126)					
	Reduced model			Full model		
	Estimate (95% CI)		*p*-value	Estimate (95% CI)		*p*-value
Intercept	0.29	(0.22–0.37)	7.5e − 12*	4.58	(3.59 – 5.58)	**4.6e − 15**
Storage as DNA	9.64e-03	(**− **1.09e** − **02–3.02e** − **02)	0.360	8.57e** − **03	(− 7.29e − 03–2.44e − 02)	0.292
Storage as buffy coat				** − **2.52e** − **06	(− 3.52e − 04–3.47e − 04)	0.989
Age				2.31e** − **07	(− 2.17e − 06–2.63e − 06)	0.851
CD8 + T				** − **4.28	(− 5.29– − 3.27)	**1.8e − 13**
CD4 + T				** − **4.15	(− 5.20– − 3.09)	**5.4e − 12**
NK cell				** − **4.42	(− 5.40– − 3.45)	**9.1e − 15**
B cell				** − **4.24	(− 5.25– − 3.23)	**3.3e − 13**
Monocytes				** − **4.48	(− 5.55– − 3.42)	**2.9e − 13**
Granulocytes				** − **4.34	(− 5.35– − 3.33)	**1.3e − 13**
Model AIC	− 420.3			** − 488.8**		
Model BIC	− 411.8			** − 457.6**		

Term	Validation cohort (n = 136)					
	Reduced model			Full model		
	Estimate (95% CI)		*p*-value	Estimate (95% CI)		*p*-value
Intercept	0.28	(0.25–0.31)	**2.8e − 36**	3.89	(2.80–4.98)	**1.5e − 10**
Storage as DNA	0.02	(0.00–0.03)	**0.030**	9.36e − 03	(− 3.01e − 03–2.17e − 02)	0.140
Age				1.42e − 06	(-1.64e − 06–4.48e − 06)	0.366
CD8 + T				− 3.65	(− 4.76– − 2.55)	**2.0e − 09**
CD4 + T				− 3.47	(− 4.61– − 2.32)	**2.5e − 08**
NK cell				− 3.60	(− 4.68– − 2.51)	**1.6e − 09**
B cell				− 3.38	(− 4.51– − 2.25)	**4.0e − 08**
Monocytes				− 3.48	(− 4.63– − 2.33)	**2.7e − 08**
Granulocytes				− 3.70	(− 4.80– − 2.60)	**1.0e − 09**
Model AIC	− 494.7			** − 558.0**		
Model BIC	− 486.0			** − 528.9**		

*Bolded *p*-values indicate *p* < 0.05

In both discovery and validation cohorts, the full models accounting for covariates: age and cell type composition for both cohorts, and storage duration as buffy coat for the discovery cohort are more suitable than the reduced models not accounting for those covariates: the Akaike information (AIC) and Bayesian information criteria (BIC) for the full models are more negative than those for the reduced models implying an optimal trade-off between model fit and model complexity. In both cohorts, there is strong evidence in the full model that the estimated compositions of cell types (monocytes, granulocytes; CD8 + T, CD4 + T, NK, and B cells) are associated with mean global methylation (*p* ≤ 4.0e-08) (Table 2). Repeating this analysis using methylation beta-values instead of M-values yields similar results, except the term for storage duration as DNA in the validation cohort’s reduced model is no longer significant at *α* = 0.05 (*p* = 0.132) (Additional file 2: Table S1).

### Differential methylation of individual CpGs

In both the discovery and validation cohorts, there was a trend of hypomethylation when each CpG was analyzed for differential methylation across increasing storage durations as DNA, even after controlling for available covariates: age and cell type compositions for both cohorts, and storage duration as buffy coat for the discovery cohort; and even after accounting for FDR via *q*-values.

In the discovery cohort, 4,049 CpGs out of the 830,545 CpGs tested (0.5%) are hypomethylated but only 50 CpGs are hypermethylated across increasing storage durations as DNA at *q*-value < 0.05 (Fig. 2C). After controlling for age, cell type composition, and storage duration as buffy coat, the bias toward hypomethylation remained with 1831 hypomethylated CpGs but only 21 hypermethylated CpGs. Contrary to the reduced and full models for global methylation where the full model including covariates was the more appropriate model by AIC and BIC; the reduced model without covariates was more appropriate to model differential methylation of individual CpGs as compared to the full model (Additional file 2: Table S2).

The same trend of hypomethylation was observed in the validation cohort. When fit with the reduced model without covariates, 63 CpGs were hypomethylated but only 6 CpGs were hypermethylated at a *q*-value < 0.05 (Fig. 2D). When fit with the full model with covariates, there were three hypomethylated CpGs and no hypermethylated CpGs at the same *q*-value cut-off. The smaller number of hypomethylated CpGs in the validation cohort could be explained by the shorter duration of storage as DNA, lower resolution of data since storage duration as DNA was recorded to the nearest week for the validation cohort but nearest day for the discovery cohort, or possibly due to the heterogeneous conditions of storage as DNA (4°C for up to two months, thereafter -20°C until library preparation; see *Methods*). As with the discovery cohort, the reduced model without covariates fits the validation cohort better than the full model with covariates (Additional file 2: Table S3). Repeating these analyses using methylation beta-values instead of M-values yields the trend of hypomethylation with storage duration as DNA (Additional file 1: Figures S1 and S2).

Since most human CpGs are methylated, it is possible that more CpGs appear hypomethylated due to technical noise from already methylated CpGs appearing hypomethylated rather than hypermethylated. To verify if this hypomethylation bias could have occurred due to the relative abundance of methylated CpGs in the human genome, the storage duration as DNA was randomly reassigned for all samples by random permutation, and then the analysis was repeated. The randomly permutated analysis yielded no differentially methylated CpGs in either cohort (Additional file 1: Figures S3 and S4), suggesting that the hypomethylation bias is not due to the relative abundance of methylated CpGs in the human genome. The smaller *q*-values in the randomly reassigned analysis for the discovery cohort (Additional file 1: Figure S3) as compared to the validation cohort (Additional file 1: Figure S4) can be explained by the larger range of methylation M-values and higher resolution of storage durations for the discovery cohort (Fig.  2 A and B).

### Genomic distribution of hypomethylated CpGs

The discovery cohort and validation cohort share more hypomethylated CpGs than expected by chance. To investigate if the identities of hypomethylated CpGs are random, or if some CpGs are more likely to be hypomethylated with storage duration as DNA than others, we first construct a list of hypomethylated CpGs with discovery *q*-value < 0.05 and validation *p*-value < 0.05 (Additional file 1: Table S4). In the validation cohort, 86,073 of the 827,646 (10.4%) CpGs tested were hypomethylated at *p* < 0.05. For the 4,049 CpGs which were hypomethylated at *q*-value < 0.05 in the discovery cohort, however, 692 (17.1%) were also hypomethylated at *p-*value < 0.05 in the discovery cohort. Thus, CpGs which are hypomethylated at discovery *q*-value < 0.05 were more likely to also be hypomethylated at validation *p*-value < 0.05 (odds ratio = 1.79 [1.64 – 1.94], *p* = 1.4e-38) than expected by chance.

To explain this observed propensity for certain CpGs to be hypomethylated, we checked if certain genomic features were over- or under-represented in the list of hypomethylated CpGs. In both the discovery (*q* < 0.05) and validation (*p* < 0.05) cohorts, we observed that CpGs closer to CpG islands have a greater tendency to be hypomethylated; except for CpGs in the CpG island themselves, which are less likely to be hypomethylated (Table 3), possibly because CpG islands are frequently already unmethylated.

**Table 3:** The relative proportions of CpGs which are hypomethylated in both discovery (*q* < 0.05) and validation (*p* < 0.05) cohorts, by genomic loci

Genomic loci	Proportion tested (%)	Proportion validated (%)	Odds ratio (95% CI)	*p*-value
Shelf, North	30056/827646 (3.6%)	19/692 (2.7%)	0.7 (0.4–1.2)	0.262
Shore, North	80282/827646 (9.7%)	114/692 (16.5%)	1.8 (1.5–2.2)*	**2.6e-8**
Island	157284/827646 (19.0%)	100/692 (14.5%)	**0.7 (0.6–0.9)**	**1.9e-3**
Shore, South	68669/827646 (8.3%)	101/692 (14.6%)	**1.9 (1.5–2.3)**	**3.8e-8**
Shelf, South	27971/827646 (3.4%)	22/692 (3.2%)	0.9 (0.6–1.4)	0.916
Open sea	463384/827646 (56.0%)	336/692 (48.6%)	**0.7 (0.6–0.9)**	**9.1e-5**

*Bolded values indicate *p* < 0.05

## Discussion

In this study, we showed that individual CpGs in extracted DNA can become hypomethylated with cryopreservation at the timescale of just a few years; even though there can be no detectable change in global methylation profiles within those same samples. Moreover, CpGs closer to CpG islands have a greater tendency to be hypomethylated in this manner, except for the CpGs in CpG islands themselves which are less likely to be hypomethylated with cryopreservation.

To the best of our knowledge, our study represents the largest analysis of the effects of long-term storage on DNA samples to date (*n* = 262). Furthermore, we show that the effects observed are consistent in both discovery (*n* = 126) and validation (*n* = 136) cohorts when analyzed separately and independently, despite the differences in their storage conditions before DNA extraction as blood/buffy coat, and after extraction as DNA. Some previous articles have observed similar patterns of hypomethylation related to cryopreservation [9, 10]. Furthermore, methylation profiles of cryopreserved and fresh DNA drawn from the same individuals clustered more by cryopreservation than by individual [14]. Another study found no change due to cryopreservation, but that could be due to the small effect size of cryopreservation on global methylation profiles (only eight loci were tested) as well as confounding due to age [11]. Conversely, researchers have found hypermethylation to be associated with cryopreservation, though as that study’s authors had noted, those changes could be due to differences in cell type composition [13]. Indeed, our analysis shows that cell type composition strongly influences global methylation profiles (*p* ≤ 4.0e-08) and the models with cell type composition have smaller AICs and BICs than models without those terms.

The mechanism for this effect likely arises from the spontaneous hydrolytic deamination of 5-methylcytosine to uracil [15]. This deamination reaction resembles that of bisulfite treatment or enzymatic conversion of unmethylated cytosines to uracils in common library preparation techniques for methylation profiling, causing originally methylated CpGs to appear unmethylated. This degradation is well-known in ancient DNA for introducing false C → T substitutions in genomic profiling [8], but its effect on the methylation profiles of more recently cryopreserved human DNA is rarely discussed [13].

It should be noted that the effect size is small, at least at the timescales we have analyzed here (maximum storage duration as DNA = 50.1 months). Indeed, at *q* < 0.05 only 4049 of the possible analyzable 830,545 (0.5%) CpGs in the discovery cohort were detectably hypomethylated. This could explain why prior studies, most of which only assessed methylation at a global scale or for a few CpGs or gene regions, reported only small or even negligible effects [16]. Nonetheless, small effects can still introduce non-trivial technical biases into downstream analyses, especially if their effect size borders on the edge of detectability as is shown here. This is especially true for sensitive techniques or “black box” machine learning algorithms which are difficult to interpret and thus difficult to check for confounders. For example, a methylation-specific PCR (MS-PCR) assay for unmethylated paternal alleles used in the diagnosis of Prader-Willi syndrome has been reported to change from hypermethylation in fresh samples to gaining some unmethylated copy of *SNRPN* gene in samples cryopreserved for only two months at − 80°C [7]. This sudden appearance of unmethylated alleles result could possibly be explained by the hypomethylation of methylated alleles, creating new targets for the MS-PCR primers. Outside of methylation analyses, deamination of cytosines in general may introduce false C → T substitutions in cancer genotyping, resulting in an overestimation of the mutational signatures resembling C → T substitutions in a CpG context.

Our study does not directly consider the effect of storage duration as blood/buffy coat, though we do account for that as a confounder in our analysis. To form a more complete view of DNA degradation in the context of biomedical research, further study is required to understand the relative effects of long-term DNA storage in different conditions, media, or buffers. The discovery and validation cohorts are different in age at blood draw, storage durations before and after extraction, and some aspects of their handling: the discovery cohort was stored as buffy coat prior to extraction while the validation cohort was not, and the validation cohort was stored at 4°C for up to two months after DNA extraction before being moved to − 20°C while the discovery cohort was stored at −20°C immediately after DNA extraction (Table 1). These cohort differences are in our opinion strengths rather than limitations of this study, as they show that our findings are generalizable across two cohorts stored according to different protocols.

## Conclusion

As laboratories and biobanks continue to accumulate cryopreserved DNA samples, there is an urgent need for new methods of DNA preservation or new computational methods to account for the effects of cryopreservation. Our study contributes to that effort by quantifying more precisely than before, the effects of cryopreservation on DNA methylation profiles. In the meantime, our study has highlighted these potential hypomethylation biases, so that future analyses can be checked for these confounders and adjusted accordingly.

## Materials and methods

### Study cohorts

This study involved 262 healthy female subjects of Chinese ancestry, with a mean age of 45 years (range: 19 to 72 years old) from three different centers – Outram SingHealth Polyclinic, Bukit Merah SingHealth Polyclinic, and KK Women’s and Children’s Hospital, Singapore. Peripheral blood samples were obtained from participants visiting Outram and Bukit Merah SingHealth Polyclinics, while DNA samples from KK Women’s and Children’s Hospital were archival samples acquired from the DNA Diagnostic and Research Laboratory. The discovery cohort of 126 subjects comprised of participants from both Outram and Bukit Merah SingHealth Polyclinics, while the validation cohort consisted of 136 subjects from KK Women’s and Children's Hospital (Fig. 1A, Table 1). Written informed consent was obtained from all participants, and the study was approved by the SingHealth Centralized Institutional Review Board (CIRB Ref: 2018/2147 and 2018/2874).

### DNA extraction

For the discovery cohort, buffy coat was isolated from peripheral blood within two hours of blood draw then stored at − 20°C. Thereafter, DNA was extracted using the QIAamp DNA Blood Kit (Qiagen, Hilden, Germany) according to the manufacturer’s instructions and stored again at -20°C. The duration of storage as buffy coat before extraction and the duration of storage as DNA after extraction was recorded in days.

For the validation cohort, samples were first stored as blood at 4°C for up to five days. Then, DNA extraction was performed using the Roche MagNA Pure Compact System (Nucleic Acid Purification) (Roche, Basel, Switzerland). Thereafter the samples were stored as extracted DNA at 4°C for up to two months. Finally, the extracted DNA was moved to archival storage at -20°C. The duration of storage as DNA after extraction was noted to the closest week.

The concentration of DNA was quantified using QuantiFluor dsDNA system (Promega, Madison, WI), and fluorescence readings at 504nm_Ex_/531nm_Em_ were measured using a 96-well plate reader (TECAN, Austin). DNA purity was assessed using a Nanodrop ND-1000 spectrophotometer (Thermo Scientific).

### DNA methylation assay

Epigenome-wide DNA methylation profiling was performed using the Infinium MethylationEPIC bead chip (Illumina, San Diego, CA), which targets more than 850,000 CpGs. A minimum of 600 ng of genomic DNA obtained from each patient was sent to Macrogen, Inc (Korea) for the EPIC microarray analysis. Briefly, the genomic DNA was bisulfite converted using the EZ DNA methylation kit (Zymo Research, Irvine, CA). The resulting bisulfite-converted DNA was then amplified, hybridized onto the EPIC bead chips, and scanned using the Illumina iScan scanner, following the standard Illumina procedures.

### Statistical analysis

CpGs were preprocessed by removing known cross-reactive probes [17], then each cohort was normalized and batch-corrected separately by functional normalization, as implemented in *R/Bioconductor* package *minfi* (preprocessFunnorm) [18–20]. Thereafter, CpGs near single nucleotide polymorphisms (SNPs) were removed, as well as CpGs with detection *p*-values greater than 0.01 in any sample. There were no obvious outliers or clusters in PCA (Additional file 1: Figures S5 and S6). Annotations for the Methylation EPIC probes and their corresponding CpGs were obtained from the *R/Bioconductor* package IlluminaHumanMethylationEPICanno.ilm10b4.hg19 version 0.6.0 [21].

The *R* package *MASS* was used to fit a robust linear regression model for global methylation and the *R* package *stats* was used to run the Fisher’s exact test [22]. The linear model for differential methylation for individual CpGs was fit using the *R/Bioconductor* package *limma* [23]. The *R* package *q-value* was used to estimate *q*-values [24]. Missing values were omitted before fitting the linear model, which is the default behavior for *limma.*

Storage durations and age were encoded in years. Cell type compositions were estimated using the *minfi* implementation of the algorithm by Houseman and colleagues [25] and encoded as proportions.

### Supplementary Information

**Additional file1: Fig. S1.** For the discovery cohort, the change in methylation beta-value per year of storage as DNA and their associated q-values. **Fig. S2.** For the validation cohort, the change in methylation beta-value per year of storage as DNA and their associated q-values. **Fig. S3.** For the discovery cohort, after randomly reassigning the storage duration as DNA of all samples by random permutation, there are no longer any CpGs achieving the q-value < 0.05 threshold. Each point shows the change in methylation M-value per year of storage as DNA and their associated q-values, and the horizontal line represents the q-value < 0.05 threshold. **Fig. S4.** For the validation cohort, after randomly reassigning the storage duration as DNA of all samples by random permutation, there are no longer any CpGs achieving the q-value < 0.05 threshold. Each point shows the change in methylation M-value per year of storage as DNA and their associated q-values, and the horizontal line represents the q-value < 0.05 threshold. **Fig. S5.** For the discovery cohort, PCA of the methylation M-values colored by the storage duration as DNA. **Fig. S6.** For the validation cohort, PCA of the methylation M-values colored by the storage duration as DNA

**Additional file2: Table S1.** Results for the robust linear regression for mean global methylation, repeated using methylation beta-values. **Table S2.** Mean AIC and BIC for the discovery cohort for different models testing differential methylation in association with duration in storage as DNA. **Table S3.** Mean AIC and BIC for the validation cohort for different models testing differential methylation in association with duration in storage as DNA. **Table S4.** CpGs hypomethylated in both the discovery (q < 0.05) and validation (p < 0.05) cohorts.

## Data Availability

The datasets used and analyzed during the current study are available from the corresponding author on reasonable request.

## Abbreviations

AIC: Akaike information
BIC: Bayesian information criteria
MS-PCR: Methylation-specific polymerase chain reaction
SNPs: Single nucleotide polymorphisms
