# Supplementary material for: Degradation of methylation signals in cryopreserved DNA
Source: Clin Epigenetics. 2023 Sep 11;15:147. doi: 10.1186/s13148-023-01565-y (PMC10496221; doi:10.1186/s13148-023-01565-y)
Supplement: Supplementary file 1 — Additional file1: Fig. S1. For the discovery cohort, the change in methylation beta-value per year of storage as DNA and their associated q-values. Fig. S2. For the validation cohort, the change in methylation beta-value per year of storage as DNA and their associated q-values. Fig. S3. For the discovery cohort, after randomly reassigning the storage duration as DNA of all samples by random permutation, there are no longer any CpGs achieving the q-value < 0.05 threshold. Each point shows the change in methylation M-value per year of storage as DNA and their associated q-values, and the horizontal line represents the q-value < 0.05 threshold. Fig. S4. For the validation cohort, after randomly reassigning the storage duration as DNA of all samples by random permutation, there are no longer any CpGs achieving the q-value < 0.05 threshold. Each point shows the change in methylation M-value per year of storage as DNA and their associated q-values, and the horizontal line represents the q-value < 0.05 threshold. Fig. S5. For the discovery cohort, PCA of the methylation M-values colored by the storage duration as DNA. Fig. S6. For the validation cohort, PCA of the methylation M-values colored by the storage duration as DNA [file 13148_2023_1565_MOESM1_ESM.docx]

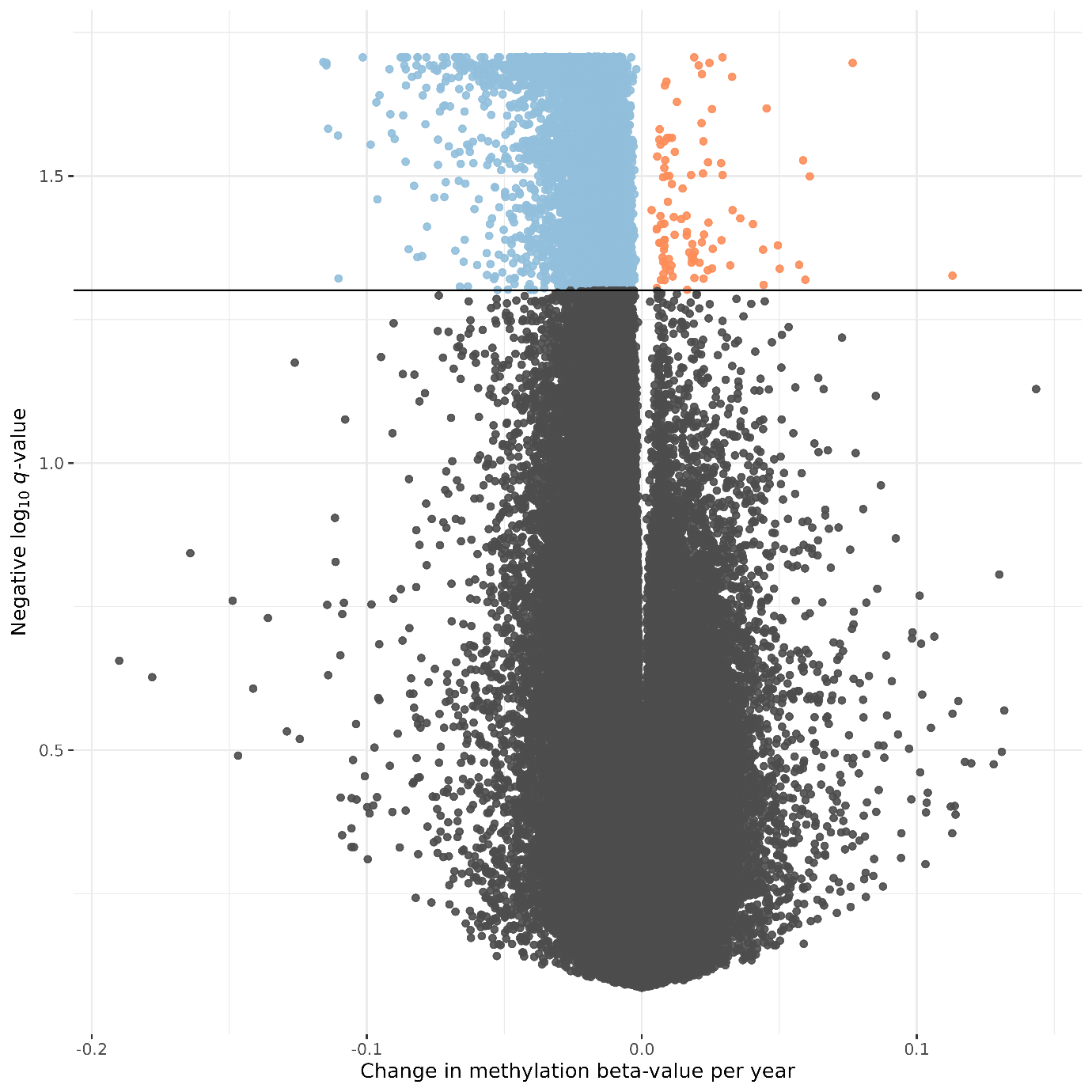


**Supplementary Figure S1.** For the discovery cohort, the change in methylation beta-value per year of storage as DNA and their associated *q*-values.


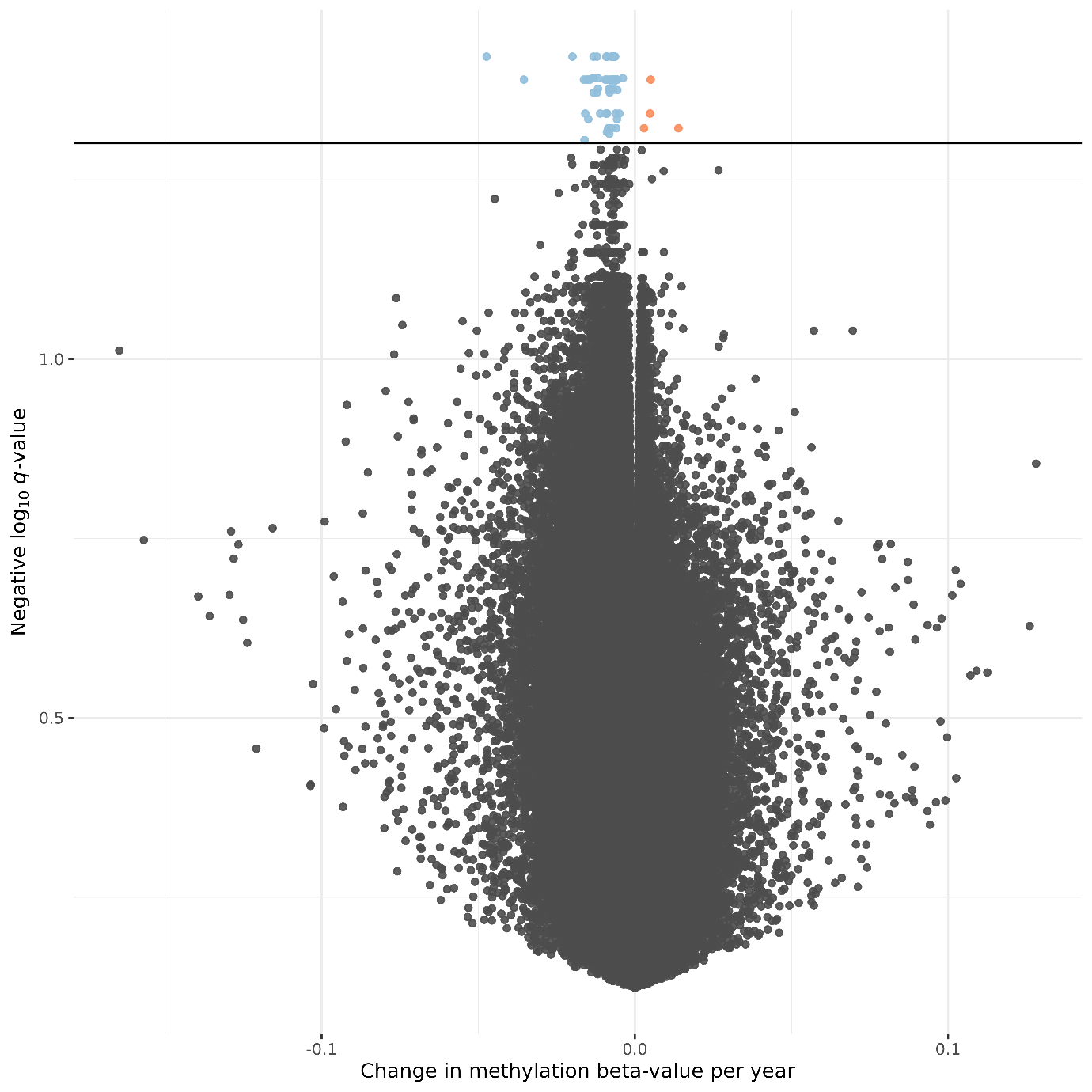


**Supplementary Figure S2.** For the validation cohort, the change in methylation beta-value per year of storage as DNA and their associated *q*-values.


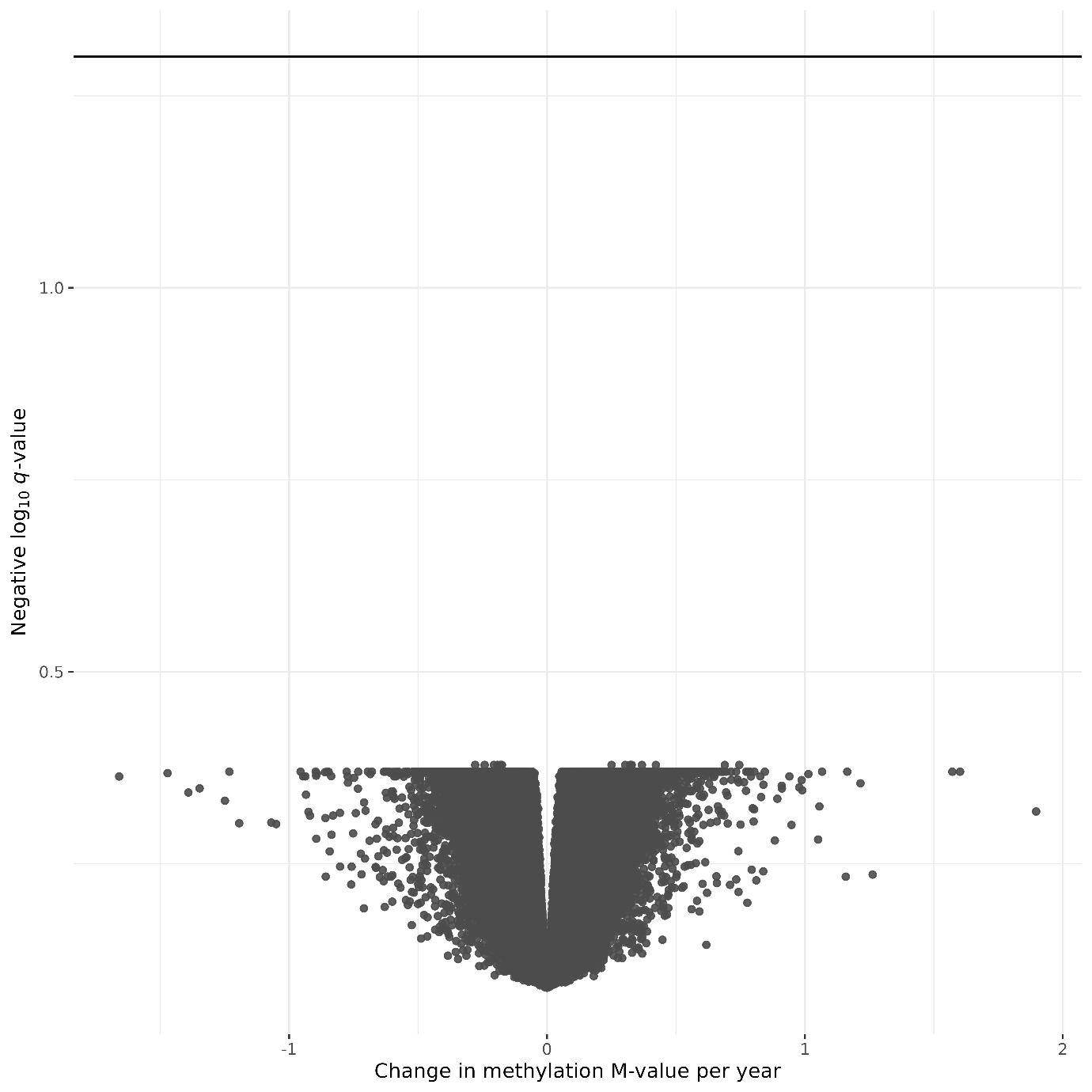


**Supplementary Figure S3.** For the discovery cohort, after randomly reassigning the storage duration as DNA of all samples by random permutation, there are no longer any CpGs achieving the *q*-value < 0.05 threshold. Each point shows the change in methylation M-value per year of storage as DNA and their associated *q*-values, and the horizontal line represents the *q*-value < 0.05 threshold.


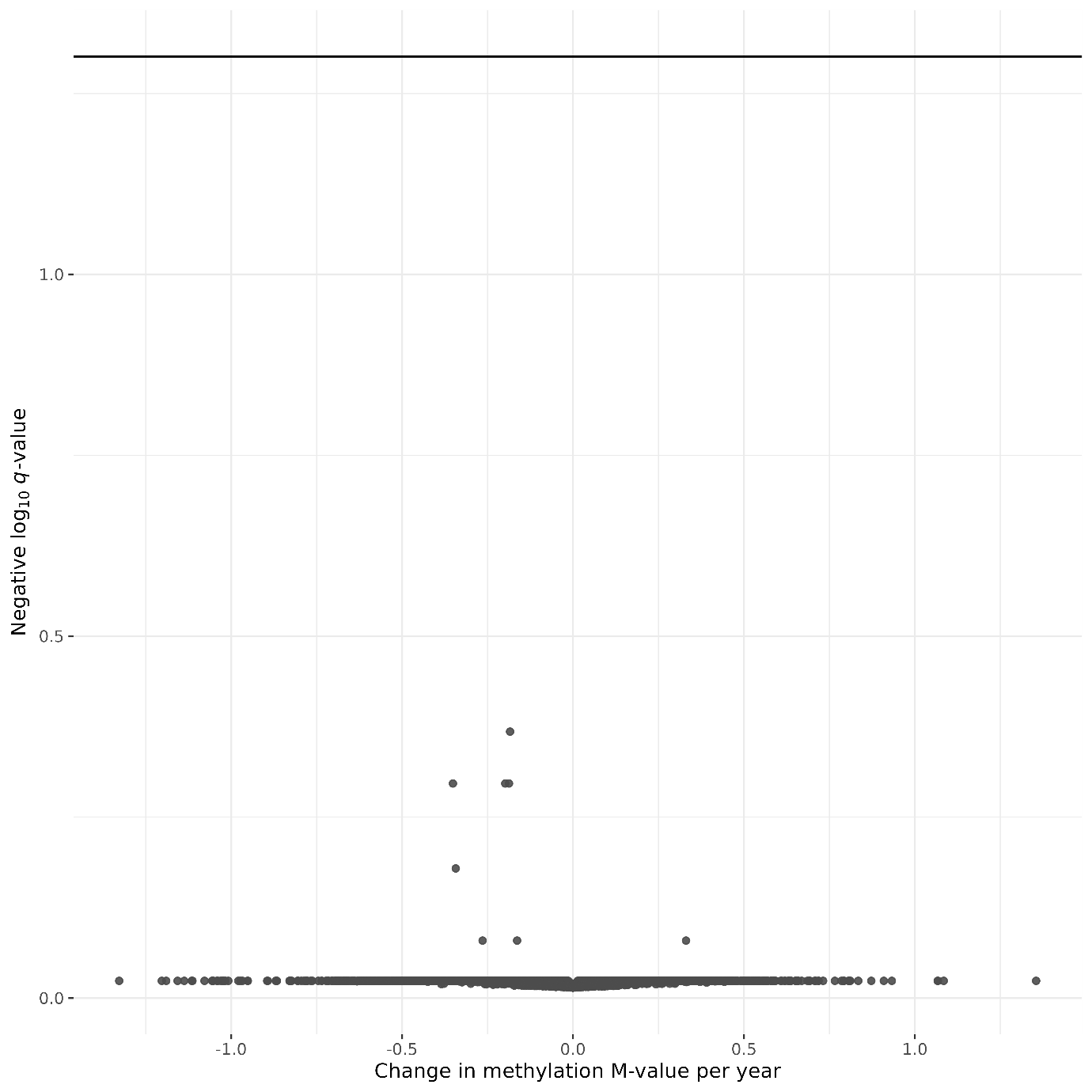


**Supplementary Figure S4.** For the validation cohort, after randomly reassigning the storage duration as DNA of all samples by random permutation, there are no longer any CpGs achieving the *q*-value < 0.05 threshold. Each point shows the change in methylation M-value per year of storage as DNA and their associated *q*-values, and the horizontal line represents the *q*-value < 0.05 threshold.

**
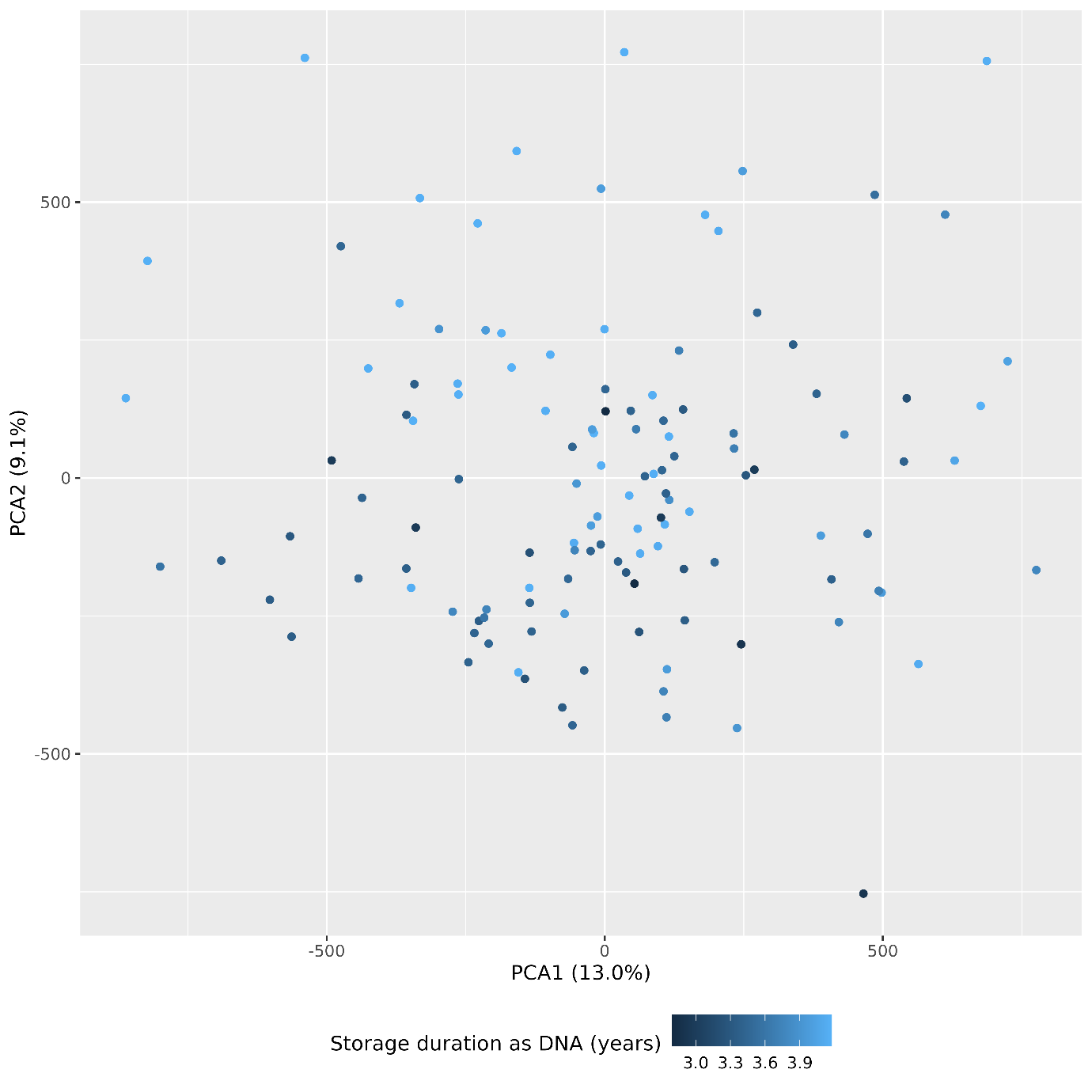
**

**Supplementary Figure S5.** For the discovery cohort, PCA of the methylation M-values colored by the storage duration as DNA.

**
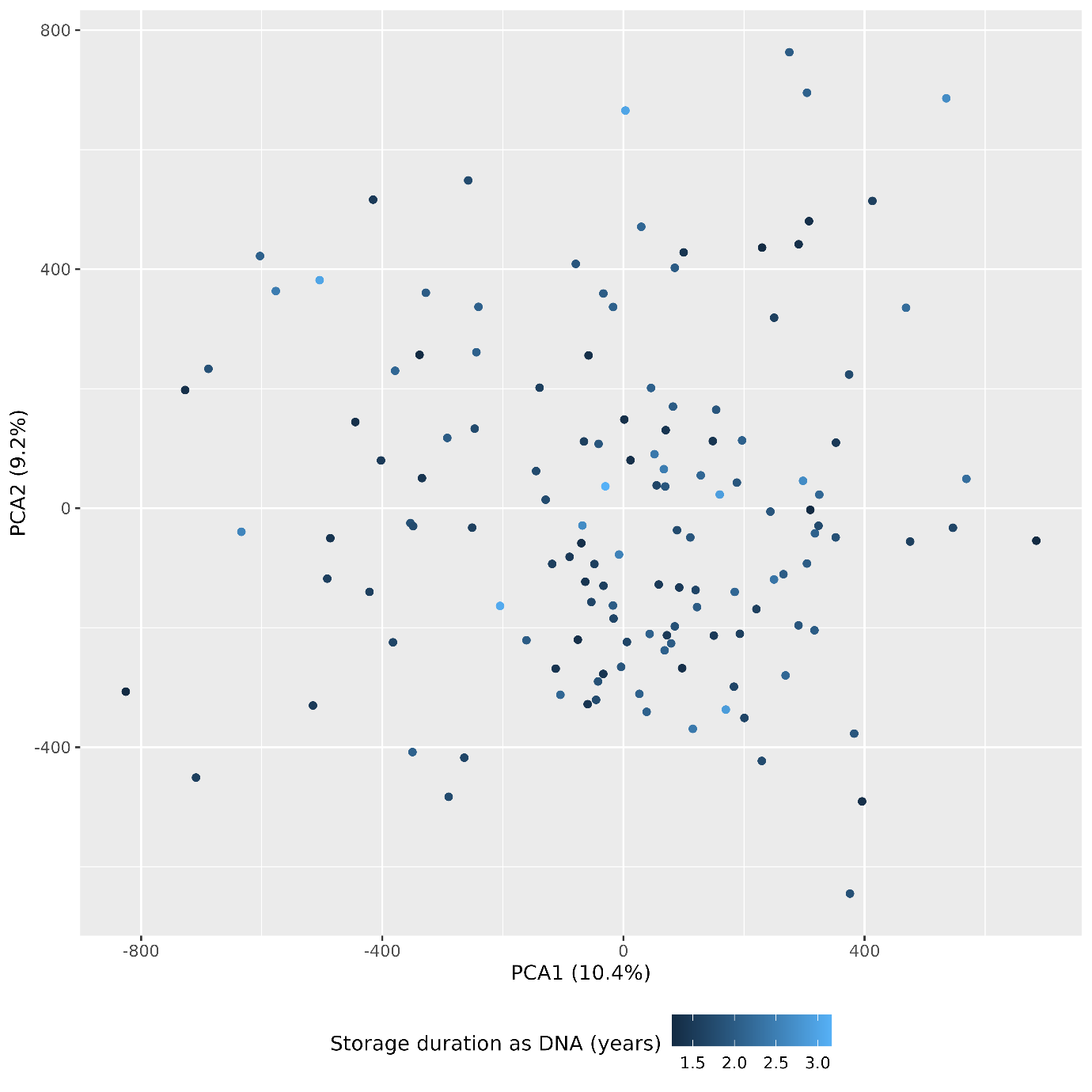
**

**Supplementary Figure S6.** For the validation cohort, PCA of the methylation M-values colored by the storage duration as DNA.
